# Obtaining the Optimal Dose in Alcohol Dependence Studies

**DOI:** 10.3389/fpsyt.2012.00100

**Published:** 2012-11-22

**Authors:** Nolan A. Wages, Lei Liu, John O’Quigley, Bankole A. Johnson

**Affiliations:** ^1^Division of Translational Research and Applied Statistics, Department of Public Health Sciences, University of VirginiaCharlottesville, VA, USA; ^2^Division of Biostatistics, Department of Preventive Medicine, Northwestern UniversityChicago, IL, USA; ^3^Université Paris VIParis, France; ^4^Department of Psychiatry and Neurobehavioral Sciences, University of VirginiaCharlottesville, VA, USA

**Keywords:** alcohol research, dose-finding, maximum tolerated dose, most successful dose, continual reassessment method

## Abstract

In alcohol dependence studies, the treatment effect at different dose levels remains to be ascertained. Establishing this effect would aid us in identifying the best dose that has satisfactory efficacy while minimizing the rate of adverse events. We advocate the use of dose-finding methodology that has been successfully implemented in the cancer and HIV settings to identify the optimal dose in a cost-effective way. Specifically, we describe the continual reassessment method (CRM), an adaptive design proposed for cancer trials to reconcile the needs of dose-finding experiments with the ethical demands of established medical practice. We are applying adaptive designs for identifying the optimal dose of medications for the first time in the context of pharmacotherapy research in alcoholism. We provide an example of a topiramate trial as an illustration of how adaptive designs can be used to locate the optimal dose in alcohol treatment trials. It is believed that the introduction of adaptive design methods will enable the development of medications for the treatment of alcohol dependence to be accelerated.

## Introduction

Alcoholism and alcohol abuse compose a large, worldwide public health problem that is responsible for significant morbidity and mortality (Mokdad et al., 2004). To this point, the most common form of treatment for alcohol dependence in the USA has been group counseling and referral to community support groups. Three medications – disulfiram, naltrexone, and acamprosate – have been approved for the treatment of alcohol dependence although the use of these medications is limited. Further, there is no single medication that is effective in every case or every person. Craving is an instrumental component of alcohol dependence and can involve a desire for the reward provided by alcohol, the need for relief from tension, or an obsessive loss of control over one’s thoughts about alcohol; hence, the most promising and efficacious medications are those that interfere with the neurotransmitters involved in craving mechanisms (Addolorato et al., 2005). The development of new and more effective medications to treat alcoholism remains a high priority (Willenbring, 2007).

Many clinical trials have been used to evaluate the efficacy and safety of new medications to treat alcoholism. Most of them involve two arms: a treatment arm and a control arm. It is often of particular interest to clinicians, however, to determine the optimal dose from a range of doses. In this case, two-arm studies are insufficient. For instance, in the single-site topiramate study (Johnson et al., 2003), topiramate’s (or matching placebo’s) dose started at 25 mg for week 1, with a 25-mg increment in weeks 2–4 and a 50-mg increment in weeks 5–8 (up to a total dose of 300 mg). The topiramate dose of 300 mg was maintained between weeks 8 and 12. A similar dose-escalating scheme was employed in the multi-site topiramate study (Johnson et al., 2007). These proof-of-concept trials established the overall topiramate treatment effect at improving drinking outcomes. However, the topiramate effect at different dose levels remains to be established so that we can identify the best dose that has the satisfactory efficacy while minimizing the rate of adverse events.

A possible solution to this problem lies in the use of an adaptive design made up of two parts. The goal of the first part would be to determine the most promising dose of topiramate and to optimize the number of patients treated at that dose level while including enough patients at neighboring doses to examine accurately the relationship. In other words, we want to locate the dose that provides the best chance for success from among a set of doses. In the second part of the design, the optimal dose found in the first stage would be compared with a placebo arm in a randomized study to establish the statistical significance of the treatment. This stage is imperative because it guards against the unlikely situation in which the optimal dose, although more efficacious than any other dose, is not more successful than placebo.

The motivation behind adaptive designs is to make use of the statistical advantages of a sequential design in combination with the ethical considerations of treating as many patients as possible at a dose believed to be the best, given prior knowledge, and accumulated data. For traditional dose-finding designs in cancer, aimed at controlling adverse events, the optimal dose is defined in correspondence to a tolerable level of toxicity, i.e., maximum tolerated dose (MTD). For designs whose aim is to identify the most successful dose (MSD), the optimal dose is the one that maximizes the overall success rate, considering both treatment benefit (efficacy) and failure. Here, failure would be defined as either unacceptable toxicity or dropout as a result of not being able to tolerate the treatment or the absence of sufficient benefit. To address the questions raised above, we can make use of the dose-finding methodology that has been used successfully in the cancer and HIV settings over the last 30 years. One such method is the continual reassessment method (CRM; O’Quigley et al., 1990), which makes use of working statistical models that have some optimal operating characteristics.

However, the implementation of adaptive designs is often challenging and is generally not readily available to practitioners. Consequently, these designs are not commonly applied in alcohol dependence trials. In this article, we will give a review of such methods and illustrate how we can apply them in the alcohol treatment field. The paper is organized as follows. In “Recent developments in dose-finding”, we give a basic introduction to the dose-finding background, mostly the original CRM to locate the MTD and MSD. In “Studies with topiramate”, we provide an example of identifying the optimal dose from a range of doses in an alcohol dependence trial. We conclude the paper with some discussion on future work.

## Recent Developments in Dose-Finding

Typically in a dose-finding (Phase 1) trial, we desire to administer as high a dose as possible without inducing too much toxicity. Usually, an increase in dose coincides with the number of patients who will experience a dose-limiting toxicity (DLT), which is typically defined by side effects that are sufficiently severe. However, an increase in dose is also accompanied by an increase in the number of patients who will benefit from the treatment. On the other hand, an absence of toxicity will be accompanied by a lack of treatment benefit. Consequently, the primary objective of a Phase 1 trial is to identify a dose with an “acceptable” toxicity rate. Furthermore, while the aim of the trial is to enhance the treatment of future patients, the best possible treatment must be administered to the patients enrolled in the study in order to adhere to certain ethical considerations. The highest dose that can be administered with an acceptable level of toxicity is the MTD.

Suppose there is a discrete set of *k* doses available, denoted *d*_1_…*d*_k_, that are ordered in terms of their probabilities of DLT, *R*(*d_i_*), at each of the levels. That is, *R*(*d_i_*) is less than *R*(*d_j_*) whenever *i* is less than *j*. In any study, the “target” dose has a probability of DLT as close as possible to some “acceptable” toxicity rate, which might typically take values 0.2, 0.25, or 0.33. A value of 0.2 means that it is acceptable for 20% of patients to have DLT. Specifically, the MTD is defined as the dose with DLT probability closest to the target rate. Based on the accumulated data, the primary objective of the study is the identification of the MTD. Consequently, estimation of the entire dose-toxicity curve is only of secondary interest in that it may aid us in locating the MTD. The CRM, proposed as a statistical design to meet the requirements of the type of studies described above, was introduced by O’Quigley et al. (1990). Information on DLT for each patient is recorded as a binary random variable with 1 indicating DLT and 0 indicating no DLT. The CRM begins by assuming a functional dose-toxicity curve to model the DLT probabilities at each dose, which, for example, could be the logistic curve or power model. This model must take values between zero and one and be monotonically increasing with dose. A comprehensive discussion of the wide variety of potential working models can be found in Shen and O’Quigley (1996). Here, we focus on the power model, which is simple and has shown itself to work well in practice. Therefore, the true probability of seeing a DLT at a given dose is $\alpha_{i}^{a}$. Here, each α*_i_* represents some initial guess for the DLT probability at dose *d_i_*, specified by the investigator prior to the beginning of the trial. O’Quigley et al. (1990) suggested that the α*_i_* be chosen to reflect prior assumptions about the DLT probabilities associated with each dose. Lee and Cheung (2009) provided a systematic approach to choosing these values. It is not expected that the working model will represent the entire dose-toxicity curve, but rather should be flexible enough to provide estimation of the dose-toxicity relationship at and around the MTD. Cheung and Chappell (2002) described the sensitivity of CRM’s operating characteristics to various choices of α*_i_*.

The method requires estimates of the probability of toxicity at the available dose levels. Using the data accumulated for each patient in the trial thus far, we can calculate the maximum likelihood estimate (MLE) of *a*, which we denote *â*. Once *â* has been found, we can obtain an estimate of the probability of toxicity at each dose level by raising α*_i_* to the power *â*. On the basis of this formula, we determine the dose given to the next entered patient to be the dose with an estimated DLT probability closest to the target toxicity rate. This process is performed after observing the response of each entered patient so that dose recommended for each patient is based on information about how well each previously entered patient tolerated the treatment. Therefore, the CRM is a sequential design that updates the DLT probability estimates and chooses the “best” treatment after each inclusion of a patient. The MTD is the recommended dose after the inclusion of a predetermined sample size of patients, which, in a typical Phase 1 trial, is around 25 patients.

## Illustration

We recall a brief example outlined in O’Quigley and Shen (1996). The simulated example investigates the operating characteristics of the CRM in a trial of 16 patients. The trial had six dose levels with true toxicity probabilities 0.03, 0.22, 0.45, 0.60, 0.80, and 0.95. The target toxicity rate was 0.20, indicating that dose level 2 is the correct MTD level with toxicity probability equal to 0.22. The initial guesses of the toxicity probability estimates were α_1_ = 0.04, α_2_ = 0.07, α_3_ = 0.20, α_4_ = 0.35, α_5_ = 0.55, α_6_ = 0.70. To be able to maximize the likelihood and generate the MLE, we need to have heterogeneity in patient observation in terms of toxicity. Thus, the trial is not considered fully underway until we have observed at least one DLT. O’Quigley and Shen (1996) advocated the use of two-stage designs with an initial escalation scheme to achieve the required heterogeneity in the responses. Some initial escalating scheme is required, and, here, we base this on a simple algorithm that includes patients in groups of three at a time. If all three remain on treatment and there is no toxicity, then the dose level is escalated. If a single subject experiences toxicity, then we remain at the same dose level. If two or more toxicities are observed, then the dose level is lowered. We continue in this way until the first DLT is encountered. As soon as we have both a toxicity and a non-toxicity, the first stage is closed; the second stage is subsequently opened, and we are in a position to fit the parameters of the under-parameterized model using the data accumulated thus far in the trial. Even though the first stage is closed, the response information accrued by the initial scheme is retained and used in the second stage.

**Figure 1:**
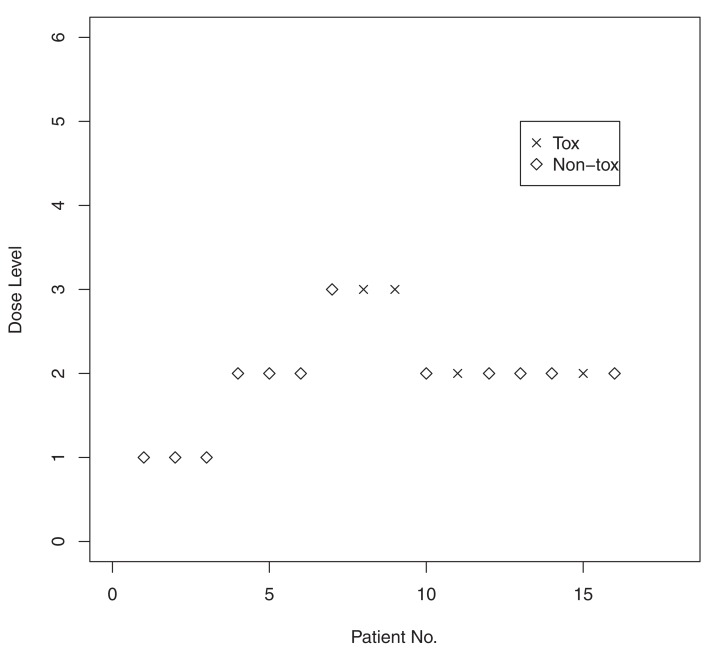
**Results from a simulated Phase I trial using the continual reassessment method**.

For example, the first three patients were entered at dose level 1, and none of the three experienced a DLT. The trial was then escalated to dose level 2, and none of the three patients entered on level 2 experienced a DLT. Escalation then took place to dose level 3, where a DLT was observed in two of the three patients. Having achieved the required heterogeneity in the responses, the MLE of *a* exists. Based on the data accumulated from the first nine entered patients, the MLE is calculated to be *â* = 0.715. It follows, from raising our α*_i_* values to this power, that we have estimated toxicity probabilities $\hat{R}$(*d*_1_) = 0.101,$\hat{R}$(*d*_2_) = 0.149,$\hat{R}$(*d*_3_) = 0.316,$\hat{R}$(*d*_4_) = 0.472,$\hat{R}$(*d*_5_) = 0.652,$\hat{R}$(*d*_6_) = 0.775. The 10th entered patient was then treated at dose level 2 because it had an estimated toxicity probability of 0.149, which was closest to our target rate of 20%. The 10th patient did not suffer a DLT, and the updated MLE for *a* becomes *â* = 0.759. Dose level 2 remained the level closest to the target level, and the 11th patient was entered at this level. The same dose was recommended to the remaining available patients so that the dose recommended as the MTD for future use after the inclusion of 16 patients was level 2. Figure 1 presents a graphical illustration of this simulated trial.

## Locating the Most Successful Dose

The original CRM design focuses only on how to locate the MTD. O’Quigley et al. (2001) proposed designs that simultaneously evaluate efficacy of treatment and toxicity in dose-finding methodology for HIV studies. The clinical setting for HIV studies allowed information on benefit to be available in a time frame similar to information on toxicity. Therefore, it was possible to evaluate overall success, which is unlike dose-finding designs for cytotoxic agents in cancer. In cancer studies, either we do not expect to observe much response or, if obtained, it is not clear how to utilize the information gathered on efficacy. Zohar and O’Quigley (2006) outlined dose-finding designs that take into account both toxicity and efficacy in the cancer setting.

## Modeling Overall Success

In this setting, we are interested in describing both safety and efficacy by two one-parameter power models (O’Quigley et al., 2001). Safety is indicated by the presence or absence of DLT, and efficacy by the presence or absence of response to the treatment. The probability of DLT at a dose level is defined as the one-parameter power model in the previous section. Similarly, the probability of response given no toxicity at dose level *d_i_* is given by $\beta_{i}^{b}$. where β*_i_* represents prior assumptions about the probabilities of response given no DLT for each of the dose levels, and *b* is a parameter to be estimated. The probability of success is given by the probability of response (efficacy) times the probability of no DLT (safety) at each dose level. The goal of the study would be to identify the dose level that maximizes the probability of success. This dose is the MSD.

Overall, the choice of the α*_i_* and β*_i_* will not have a very strong effect on operating characteristics. For large samples and under very broad conditions, the choice will have no influence at all. For finite, typically small, samples, often encountered in dose-finding studies, there will be an impact. A reasonable choice is to divide the interval (0,1) into equivalent segments. For instance, for four dose levels, both the α*_i_* and the β*_i_* could be chosen to be 0.2, 0.4, 0.6, and 0.8. These values remain invariant to power transformations. For example, the operating characteristics for small samples based on the above model are exactly identical to the square of the model, i.e., taking α*_i_* and β*_i_* to be equal to 0.04, 0.16, 0.36, and 0.64. The next entered patient will be included at the dose level *d_i_* that maximizes the estimated probability of success.

## Dose Allocation

As discussed above, a requirement to be able to estimate the parameters is that we have heterogeneity in patient observation in terms of toxicity and response. Thus, the trial is not considered fully underway until we have observed at least one toxicity and one response. This can be done in two stages. As soon as we have both a positive response and a dropout or toxicity, the first stage is closed; the second stage is subsequently opened, and we are in a position to fit the models using the data accumulated thus far in the trial. We are able to reassess the working dose-toxicity and dose-response relationships through the estimated parameters, and calculate the probability of success for each dose level. The optimal dose for the next patient will be identified as that with the best probability of success. Repeating this procedure for all the remaining subjects will yield the final optimal dose: the MSD.

## Studies with Topiramate

In this section, we will take the topiramate trial as an example to illustrate how CRM can be used to locate the MSD in alcohol treatment trials. Although topiramate has been shown to be effective at reducing drinking for alcoholics in two proof-of-concept trials (Johnson et al., 2003, 2007), the optimal dose has not be established. In the double-blind, randomized, placebo-controlled, 14-week clinical trial (Johnson et al., 2007), six doses were adopted in the dose-escalating scheme (in mg/day): 25, 50, 100, 150, 200, and 300. Suppose we wanted to choose the optimal dose in consideration of both efficacy and safety among the six doses.

The efficacy outcome of interest is the percentage of subjects with no heavy drinking days (PSNHDDs), an efficacy endpoint recommended by the Food and Drug Administration (Falk et al., 2010). It is a dichotomous endpoint measuring the frequency of heavy drinking days, whereby 0 heavy drinking days (e.g., during the last month of follow-up) is considered a good outcome and one or more heavy drinking days is considered risky drinking or a poor outcome. We also are interested in the safety measure, i.e., the adverse events. Johnson et al. (2007) found that 34 of 183 who received topiramate had adverse events. We expect that a higher dosage of topiramate is associated with a higher rate of adverse events. One of these levels will offer the greatest chance of overall success, and our purpose is to identify this level. Initially, we will treat the first cohort of patients at 25 mg/day. The dose will be increased or decreased in accordance with observations on tolerance and efficacy. Once we are able to fit the stochastic models for the rates as a function of dosage, we will use CRM-type designs to concentrate treatment on the level corresponding to the optimal level.

On the basis of our fitted model, which will enable us to provide estimates of both the probabilities of success and those of failure (failure being unacceptable toxicity, dropout, or inadequate efficacy), we will take the statistical product of the conditional probability of success multiplied by one minus the probability of treatment failure. These products will vary with dose, and the one that provides the greatest value is the one that is estimated to have the greatest probability of overall success. This level is then designated the optimal one for topiramate. This optimal level accounts for dropout, loss to follow-up, unacceptable toxicity, and true efficacy.

If the optimal level is identified before all patients have been included, then this first part of the study can be brought to an early close and the second part of the study, involving comparison with a placebo, can begin. In the second part, we could perform a double-blind trial in which alcohol-dependent patients will be randomized to either the optimal dose of topiramate found in the first part or a placebo. Applying dose-finding designs in the context of pharmacotherapy research in alcoholism could be crucial to the success of a study since an incorrectly defined dose for the second part could lead us to conclude that the treatment is ineffective, either because the treatment is not well tolerated or because it fails to deliver enough of the drug to be effective.

## Conclusion

Determining the optimal dose is of considerable clinical and scientific impact because it will enhance the safety, tolerability, and generalizability of its use. An important advantage of introducing this new methodology into the pharmacotherapy field for alcoholism is that it is more economical of time, resources, and human subjects compared with the traditional method of testing multiple fixed doses using a factorial design. Determining the optimal dose of topiramate will have an important effect on the clinical care of alcohol-dependent patients. It will widen considerably the population of those who can be provided with topiramate, enable those on the medication to derive the maximum benefit with a more tolerable adverse event profile, and improve medication compliance.

For simplicity, we make the assumption that we are dealing with a homogeneous group regarding the probability of both success and treatment failure. However, realistically, it is likely that there is some significant heterogeneity in the responses. The models described above have been extended to deal with heterogeneity, and we will be keeping this in mind as the study progresses. If we are able to identify significant sources of heterogeneity, it will be possible, within the context of a single study, to obtain more than a single successful dose, each one corresponding to a particular prognostic group (O’Quigley and Paoletti, 2003). The cost of this is a higher sample size, typically a sample of around 20% more than that encountered in the case of homogeneity.

Another question lies in clinical trials for a combination of different medications. For example, in the Combined Pharmacotherapies and Behavioral Interventions for Alcohol Dependence (COMBINE) study, a 2 (acamprosate/placebo) × 2 (naltrexone/placebo) × 2 [Cognitive Behavioral Intervention (CBI)/no CBI] factorial design was used, and a ninth group received CBI alone (Anton et al., 2006). In an ongoing trial, we are interested in the combination of ondansetron and naltrexone. Each medication has three dose levels: zero, low, and high. So, altogether, we have nine arms. Finding the best dose combination for these trials entails a large sample size, which could be prohibitive in terms of both time and money. Further, since there are two components, the ordering of the combinations, in terms of probabilities of toxicity and/or dropout and the probability of seeing a successful reduction in alcohol use, may not be fully known. For instance, it is not possible, *a priori*, to determine the toxicity order between the combination of low-dose ondansetron and high-dose naltrexone vs. that of high-dose ondansetron and low-dose naltrexone. Consequently, standard adaptive dose-finding methods may fail in this situation. To deal with this partial ordering, we could make use of a CRM design for partial orders proposed by Wages et al. (2011).

Another specific problem arising in the context of alcohol studies is that the recorded information can be subject to errors. We will, however, pay particular attention to robustness. That is, we want to know how changes in the recorded data influence the recommendations that are based on models. There are two main directions to this problem. The first is robustness of the choice of working model. It is important to underline our purpose here, which is not to see how well any given model identifies the true MSD. This problem has been studied in several simulation studies. Even had we, by good fortune, selected exactly the model that generates the observations, then, as a result of the inherent randomness in the experiment, the observed toxicity rate could have led us to recommend an incorrect level. What we wish to know is whether a different choice of model would have led us to this same recommendation, whether correct or not. It is thus important to separate out the task of identifying the correct level from that of verifying whether an identified level, under some model assumption, would remain the same under a different model assumption. Second, we need to deal with errors following model specification. Since toxicities themselves can be recorded with errors, it is important to investigate the influence of these errors on final recommendations. Sensitivity analysis could be used to find out how the recording error can impact the final decision of optimal dose. More work is needed to tackle both robustness issues simultaneously.

## Conflict of Interest Statement

Prof. Johnson has served as a consultant to Johnson & Johnson (Ortho-McNeil Janssen Scientific Affairs, LLC), Transcept Pharmaceuticals, Inc., D&A Pharma, Organon, ADial Pharmaceuticals, LLC (with which he also serves as Chairman), Psychological Education Publishing Company (PEPCo), LLC, and Eli Lilly and Company. Drs. Wages, Liu, and O’Quigley report no biomedical financial interests or potential conflicts of interest.
